# Chronic Alcohol Exposure Induced Neuroapoptosis: Diminishing Effect of Ethyl Acetate Fraction from *Aralia elata*

**DOI:** 10.1155/2019/7849876

**Published:** 2019-05-09

**Authors:** Bong Seok Kwon, Jong Min Kim, Seon Kyeong Park, Jin Yong Kang, Jeong Eun Kang, Chang Jun Lee, Sang Hyun Park, Su Bin Park, Seul Ki Yoo, Uk Lee, Dae-Ok Kim, Ho Jin Heo

**Affiliations:** ^1^Division of Applied Life Science (BK21 Plus), Institute of Agriculture and Life Science, Gyeongsang National University, Jinju 52828, Republic of Korea; ^2^Division of Special Purpose Tree, National Institute of Forest Science, Suwon 16631, Republic of Korea; ^3^Department of Food Science and Biotechnology, Kyung Hee University, Yongin 17104, Republic of Korea

## Abstract

An ethyl acetate fraction from *Aralia elata* (AEEF) was investigated to confirm its neuronal cell protective effect on ethanol-induced cytotoxicity in MC-IXC cells and its ameliorating effect on neurodegeneration in chronic alcohol-induced mice. The neuroprotective effect was examined by methylthiazolyldiphenyl-tetrazolium bromide (MTT) and 2′,7′-dichlorodihydrofluorescein diacetate (DCF-DA) assays. As a result, AEEF reduced alcohol-induced cytotoxicity and oxidative stress. To evaluate the improvement of learning, memory ability, and spatial cognition, Y-maze, passive avoidance, and Morris water maze tests were conducted. The AEEF groups showed an alleviation of the decrease in cognitive function in alcohol-treated mice. Then, malondialdehyde (MDA) levels and the superoxide dismutase (SOD) content were measured to evaluate the antioxidant effect of AEEF in the brain tissue. Treatment with AEEF showed a considerable ameliorating effect on biomarkers such as SOD and MDA content in alcohol-induced mice. To assess the cerebral cholinergic system involved in neuronal signaling, acetylcholinesterase (AChE) activity and acetylcholine (ACh) content were measured. The AEEF groups showed increased ACh levels and decreased AChE activities. In addition, AEEF prevented alcohol-induced neuronal apoptosis *via* improvement of mitochondrial activity, including reactive oxygen species levels, mitochondrial membrane potential, and adenosine triphosphate content. AEEF inhibited apoptotic signals by regulating phosphorylated c-Jun N-terminal kinases (*p*-JNK), phosphorylated protein kinase B (*p*-Akt), Bcl-2-associated X protein (BAX), and phosphorylated Tau (*p*-Tau). Finally, the bioactive compounds of AEEF were identified as caffeoylquinic acid (CQA), 3,5-dicaffeoylquinic acid (3,5-diCQA), and chikusetsusaponin IVa using the UPLC-Q-TOF-MS system.

## 1. Introduction

Alcohol has been reported to cause various diseases such as fatty liver, liver cirrhosis, cardiovascular disease, cancer of various organs, and especially damage to the brain tissue [1, 2]. In addition, alcohol can cause various neurological diseases such as Alzheimer's disease, stroke, and Korsakoff's syndrome related to thiamine deficiency [3]. For the mechanism of alcoholic neurodegeneration, it was reported that alcohol has a direct toxic effect on the brain [4]. Alcohol is dehydrogenated to acetaldehyde, which is also toxic in the central nervous system, by alcohol dehydrogenase. This acetaldehyde is then decomposed to acetate by acetaldehyde dehydrogenase [5]. Although the body has an oxidative stress defense system such as superoxide dismutase (SOD), not only does the metabolic process of alcohol produce excessive reactive oxygen species (ROS) but also chronic alcohol consumption causes an imbalance in the oxidative stress defense system [6]. As a result, oxidative stress causes secondary problems such as lipid peroxidation and mitochondria dysfunction, which lead to neuronal apoptosis in chronic alcohol-induced cognitive disorder [7]. In addition, alcohol leads to inflammatory reaction. The consumption of alcohol induces bacterial overgrowth and increases the serum concentration of the lipopolysaccharide (LPS) derived from the cell walls of intestinal gram negative-bacteria [8]. Increased LPS in the blood induces inflammatory cytokines from Kupffer cells located in the liver [9]. The inflammatory cytokines activate microglial cells in the brain that are responsible for brain immunity, intensifying the inflammatory response. Continuous stimulation of the microglia leads to neuronal inflammation, which ultimately leads to the apoptosis of brain cells [10].

*Aralia elata* grows in the wild in tropical areas, and its stems and root bark are widely used for various diseases, such as diabetes, hypotension, and hepatitis, as folk medicine in East Asia [11]. *A. elata* contains various saponins such as stipuleanosides, elatoside, and chikusetsusaponin IVa [12]. Several previous investigations reported that *A. elata* has various bioactivities, such as an anti-inflammatory effect, antioxidant activity, and anti-fatty liver effect [13–15]. However, there are few studies about the inhibitory effects on alcohol-induced neurodegeneration and cognitive deficit. Therefore, our research was designed to study the use of *A. elata* for the improvement of ethanol-induced cognitive dysfunction and neurodegeneration, and the major bioactive compounds with ameliorating effects were identified.

## 2. Materials and Methods

### 2.1. Materials

Minimum essential medium (MEM) media and fetal bovine serum (FBS) were purchased from Gibco-BRL Co. (Grand Island, NY, USA). Penicillin, streptomycin, methylthiazolyldiphenyl-tetrazolium bromide (MTT), 2′,7′-dichlorodihydrofluorescein diacetate (DCF-DA), vitamin C, H_2_O_2_, ethanol, acetylthiocholine, 5,5′-dithiobis (2-nitrobenzoic acid) (DTNB), trichloroacetic acid, thiobarbituric acid, bovine serum albumin, dimethyl sulfoxide (DMSO), egtazic acid (EGTA), and 5,5′,6,6′-tetrachloro-1,1′,3,3′-tetraethylbenzimidazolocarbocyanine iodide (JC-1) were purchased from Sigma-Aldrich Chemical Co. (St. Louis, MO, USA). An ENLITEN adenosine triphosphate (ATP) assay system was purchased from Promega Corp. (Madison, WI, USA). ProtinEX Animal cell/tissue, a tissue lysis buffer, was purchased from GeneAll Biotechnology (Seoul, Korea). Phosphorylated c-Jun N-terminal kinases (*p*-JNK), phosphorylated Tau (*p*-Tau), and *β*-actin were purchased from Santa Cruz Biotechnology (Dallas, TX, USA) and phosphorylated protein kinase B (*p*-Akt) and Bcl-2-associated X protein (BAX) were purchased from Cell Signaling Technology (Danvers, MA, USA).

### 2.2. Sample Preparation

*A. elata* was purchased from Changnyeong in Korea, and washed *A. elata* was lyophilized using a freeze-drying apparatus (OPERON, Gimpo, Korea) and ground into powder form. This sample was stored at -20°C until use. The sample was extracted with 80% ethanol at 40°C for 2 h. The extract was filtered using filter paper (Whatman International Limited, Kent, UK), and the extractive solvent was completely removed using a vacuum evaporator (N-N series, EYELA Co., Tokyo, Japan). Then, the sample was mixed with distilled water, and it was fractionated using *n*-hexane, chloroform, and ethyl acetate of the same volume, continuously. Fractionates were lyophilized, and the ultimate ethyl acetate fraction of *A. elata* (AEEF) was used as a sample in the preliminary study.

### 2.3. Measurement of Oxidative Stress Levels and Cell Viability in MC-IXC Cells

#### 2.3.1. Cell Culture

MC-IXC cells, a neuronal cell line (CRL-2270, American Type Culture Collection, Rockville, MD, USA) were cultured in MEM media containing 10% FBS, 50 unit/mL penicillin, and 100 *μ*g/mL streptomycin. The cells were incubated in an incubator maintained at 5% CO_2_ and 37°C.

#### 2.3.2. Reactive Oxygen Species (ROS)

The level of ROS was determined by the DCF-DA method [16]. Cells seeded as 1 × 10^4^ cells/well (*n* = 5) in a 96-well black plate were incubated for 24 h. Vitamin C as a positive control or different concentrations of AEEF were treated to stabilize the seeded cells for 24 h. To confirm the protective effect against H_2_O_2_, every well except for the control was treated with 200 *μ*M H_2_O_2_ for 3 h. Also, to investigate the protective effect against ethanol, every well except for the control was provided with 500 mM ethanol for 24 h after sample treatment for 3 h. Finally, 50 *μ*M DCF-DA in phosphate buffered saline (PBS) was treated in every well for 50 min to measure intracellular oxidative stress. The level of ROS was measured using a fluorescence microplate reader (Infinite 200, Tecan Co., Zurich, Switzerland) at 485 nm excitation and 530 nm emission filters.

#### 2.3.3. Cell Viability

Cell viability was measured by the MTT method [16]. Cells were seeded and pretreated with EFAE or vitamin C in the same manner as the DCF-DA method in a 96-well plate. As a final step, MTT reagent was treated in every well for 3 h, and the formed formazan was dissolved by DMSO. Then, cell viability was measured using a microplate reader (Epoch 2, BioTek Instruments Inc., Winooski, VT, USA) with a wavelength of 570 nm (reference wavelength of 690 nm).

### 2.4. Animal Experimental Design

**Figure 1:**
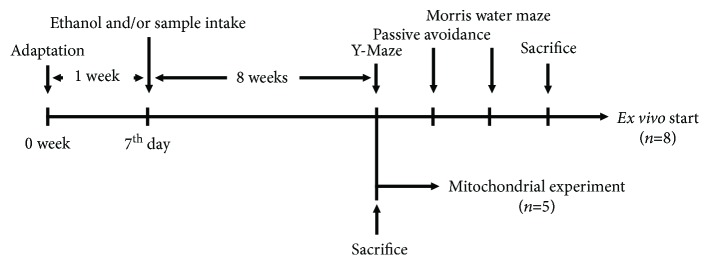
Experimental design of the *in vivo* test for alcohol-induced learning and memory impairment in mice.

C57BL/6 mice (male, 4 weeks) were purchased from Samtako (Osan, Korea). These mice were housed under standard laboratory conditions and were acclimatized to conditions for one week. The experimental groups were divided as follows: a control group administered water (orally, *n* = 13), alcohol group administered ethanol (orally, 25% *v*/*v*; 5 g/kg of body weight, *n* = 13), AEEF 50 group administered ethanol and AEEF 50 mg/kg of body weight (orally, *n* = 13), and AEEF 100 group administered ethanol and AEEF 100 mg/kg of body weight (orally, *n* = 13) for 8 weeks (Figure 1) [17–19]. All animal experiments complied with the guidelines of the Ethical Committee of the Ministry of Health and Welfare, Korea, and experimental protocols were approved by the institutional Animal Care and Use Committee (IACUC) of Gyeongsang National University (certificate: GNU-150226-M0004). After the administration of alcohol and sample was completed, the experiment was divided into two groups. One group performed cognitive *in vivo* tests and sacrificed for *ex vivo* experiments (*n* = 8). The other group was immediately sacrificed for mitochondrial-related experiments after administration of alcohol and sample (*n* = 5).

### 2.5. Behavioral Tests

#### 2.5.1. Y-Maze Tests

Space perception ability was assessed using a Y-maze test. The Y-maze consists of three equal angle arms 33 cm long, 10 cm wide, and 15 cm high. Each mouse was allowed to freely move in the Y-maze for 8 min, and the movement was recorded and analyzed using Smart 3.0 software (Panlab, Barcelona, Spain). Alternation behavior was calculated using the following equation: alternation behavior (%) = number of spontaneous alternation/(total arm entries − 2) × 100 [20].

#### 2.5.2. Passive Avoidance Tests

Learning ability and short-term memory were confirmed using a passive avoidance test [20]. The experimental apparatus for passive avoidance was composed in two areas with adjustable brightness separated by a door. Mice were identically placed in the bright area. When the mouse stepped into the dark area, the door closed. Then, a 0.5 mA electric shock was immediately applied to the mouse for 3 sec through steel rods. After 24 h, the mice were placed in the bright area again, and the time they remained in the bright area was recorded up to 300 sec.

#### 2.5.3. Morris Water Maze Tests

To evaluate cognitive memory and spatial learning ability, a Morris water maze test was conducted [21]. The Morris water maze consists of a round pool (120 cm in diameter and 50 cm in height) filled with water mixed with milk powder, at 20-22°C. The pool was divided into four quadrants, W, E, S, and N zones. A white platform was located in the N zone. During learning trials for 4 days, mice freely swam for 60 sec to find the hidden platform. The mice that found the platform on their own remained for 10 sec. When the mice failed to find it, they were moved to the platform and stayed for 20 sec. The movement to search for the platform was recorded using Smart 3.0 software (Panlab). After the training period, each mouse freely swam in the pool for 90 sec without the platform as a probe test and the retention time in the N zone was recorded to confirm long-term memory and learning ability.

### 2.6. Preprocessing of the Brain Tissue for Biochemical Assays

After the *in vivo* tests, the mice were fasted for 12 h and sacrificed for *ex vivo* experiments. Brains were collected and kept at -70°C until use. To investigate the biomarkers, the brains were homogenized with 10-fold PBS using a bullet blender (Next Advance Inc., Averill Park, NY, USA) at ice-cold temperatures. Then, homogenates were centrifuged in the appropriate conditions for each experiment.

### 2.7. Determination of the Antioxidant System

#### 2.7.1. Determination of Malondialdehyde (MDA) Content

The homogenized brain tissue was centrifuged at 2,450 g for 10 min at 4°C. The obtained supernatants were mixed with 1% phosphoric acid and 0.67% thiobarbituric acid. The mixture was boiled for 1 h at 95°C and was measured by spectrophotometer (Libra S32PC, Biochrom Ltd., Cambridge, UK) at 532 nm. The absorbance values were substituted into the MDA standard curve to calculate MDA content, and the MDA content was expressed as MDA content per protein level measured by the Bradford method [22].

#### 2.7.2. Determination of Superoxide Dismutase (SOD) Content

The homogenized brain tissue was centrifuged at 400 g for 10 min at 4°C, The obtained pellets were mixed with extractive buffer (10x SOD buffer solution of assay kit, 20% (*v*/*v*) Triton x-100, and 200 mM phenylmethanesulfonyl fluoride) and resuspended. After 30 min, the mixtures were centrifuged at 10,000 g for 10 min and the supernatants were finally obtained for a SOD assay. The SOD assay was conducted according to the manufacturer's process and measured using a microplate spectrophotometer (Epoch 2) at 450 nm. The absorbance values were substituted into the SOD standard curve to calculate SOD content.

### 2.8. Cholinergic System Evaluation

#### 2.8.1. Determination of Acetylcholine (ACh) Content

To evaluate AChE activity and ACh content, the homogenate was centrifuged at 14,000 g for 30 min at 4°C. To assess ACh content, alkaline hydroxylamine reagent was added to the obtained supernatants, which consisted of 2 M hydroxylammonium chloride and 3.5 M sodium hydroxide at the same volume. Then, 0.5 N HCl and 0.37 M FeCl_3_·6H_2_O were reacted, and the mixture was measured using a microplate spectrophotometer (Epoch 2) at 540 nm. The absorbance values were substituted into the ACh standard curve, which was drawn using acetylcholine chloride in 1 mM sodium acetate trihydrate (pH 4.5).

#### 2.8.2. Determination of Acetylcholinesterase (AChE) Activity

To assess AChE activity, 50 mM of sodium phosphate buffer (pH 8.0) was added to the obtained supernatants and this mixture was incubated for 15 min at 37°C. Then, 500 *μ*M substrate solution (500 *μ*M acetylthiocholine and 1 mM DTNB in buffer) was added, and absorbance was measured using a microplate spectrophotometer (Epoch 2) at 405 nm.

### 2.9. Mitochondrial Activities

#### 2.9.1. Isolation of Mitochondria

The brain tissue was homogenized with a 10-fold isolation buffer (215 mM mannitol, 75 mM sucrose, 0.1% BSA, 20 mM HEPES sodium salt, and 1 mM EGTA; pH 7.2) using a bullet blender (Next Advance Inc.). The homogenates were centrifuged at 1,300 g for 10 min to remove the unbroken cells. Then, the supernatant was centrifuged again at 13,000 g for 10 min, and the supernatants were removed to obtain a pellet. The obtained pellet was resuspended in the isolation buffer with 0.1% digitonin. After 5 min, the mixture was centrifuged at 13,000 g for 15 min. Then, buffer was added to the pellet without EGTA and as a final step spun down again at 10,000 g for 10 min to obtain mitochondria. The mitochondrial pellet was reacted with buffer without EGTA and used for each experiment [23].

#### 2.9.2. ROS Measurement

Mitochondrial ROS was determined using the DCF-DA method [16]. The isolated mitochondria were reacted with 25 *μ*M DCF-DA for 25 min. Fluorescence was measured using a fluorescence microplate reader (Infinite 200).

#### 2.9.3. MMP Measurement

To measure mitochondrial membrane potential (MMP), the isolated mitochondria was diluted to the same protein concentration using the buffer without EGTA and 5 mM pyruvate, 5 mM malate, and 1 *μ*M JC-1 were added to the buffer. The mixture was incubated for 20 min and measured with a fluorescence microplate reader (Infinite 200) at 530 nm (excitation filters) and 590 nm (emission filters).

#### 2.9.4. ATP Measurement

The measurement of the ATP content of isolated mitochondria was conducted with an ATP bioluminescence assay kit in accordance with the manufacturer's process and measured using a luminescence microplate reader (GloMax-Multi+ Detection System, Promega Corp., Madison, WI, USA). The ATP content was calculated using a standard curve.

### 2.10. Western Blot Analysis

The brain tissue of the experimental animals (*n* = 3) was homogenized to extract protein using a bullet blender (Next Advance Inc.) with ProtinEX Animal cell/tissue containing a 1% protease inhibitor cocktail (PPI-1015, Quartett, Berlin, Germany). This homogenized brain tissue was centrifuged at 13,000 g for 15 min. The protein samples were loaded with the same concentration of protein and separated by SDS-PAGE gel. Then, the protein was transferred to a polyvinylidene difluoride membrane. The membrane was blocked with 5% skim milk in TBST buffer (20 mM Tris-HCl, 137 mM sodium chloride, and 0.1% Tween 20; pH 7.6) for 1 h. The blocked membrane was reacted in diluted (1 : 1,000) primary antibodies including *p*-JNK, *p*-Akt, BAX, *p*-Tau, and *β*-actin and gently shaken overnight. Then, the membrane was washed using TBST and soaked in a secondary antibody for 1 h. The membrane was then completely washed with TBST. Finally, the level of protein was detected using TMB solution (Thermo Scientific, Rockford, IL, USA). Band images were obtained using an Epson scanner (L655 series, Epson, Suwa, Japan), and the image was analyzed using ImageJ (National Institutes of Health, Bethesda, MD, USA).

### 2.11. Analysis of Bioactive Compounds

The main bioactive compounds of AEEF were analyzed using ultraperformance liquid chromatography/quadrupole time-of-flight tandem mass spectrometry (UPLC-Q-TOF-MS, Vion, Waters Corp., Milford, MA, USA). The AEEF dissolved in methanol was eluted by linear gradient of acetonitrile for 8 min and dwindled acetonitrile for 8-10 min with a flow rate of 0.4 mL/min using a C_18_ column (100 × 2.1 mm, 1.7 *μ*m, Waters Corp.). The physiological compounds were analyzed by negative electrospray ion (ESI) mode to gain MS data. The conditions used for the ESI source were as follows: ramp collision energy, 20–45 V; oven temperature, 40°C; capillary voltage, 3 kV; and mass range, 50–1200 *m*/*z*.

### 2.12. Statistical Analysis

Result expression was presented as mean and standard deviation (SD). The statistical relation of groups was verified by one-way analysis of variance with Duncan's new multiple-range test (*p* < 0.05) with SAS 9.4 (SAS Institute Inc., Cary, NC, USA).

## 3. Results

### 3.1. Neuronal Cell Protective Effect

**Figure 2:**
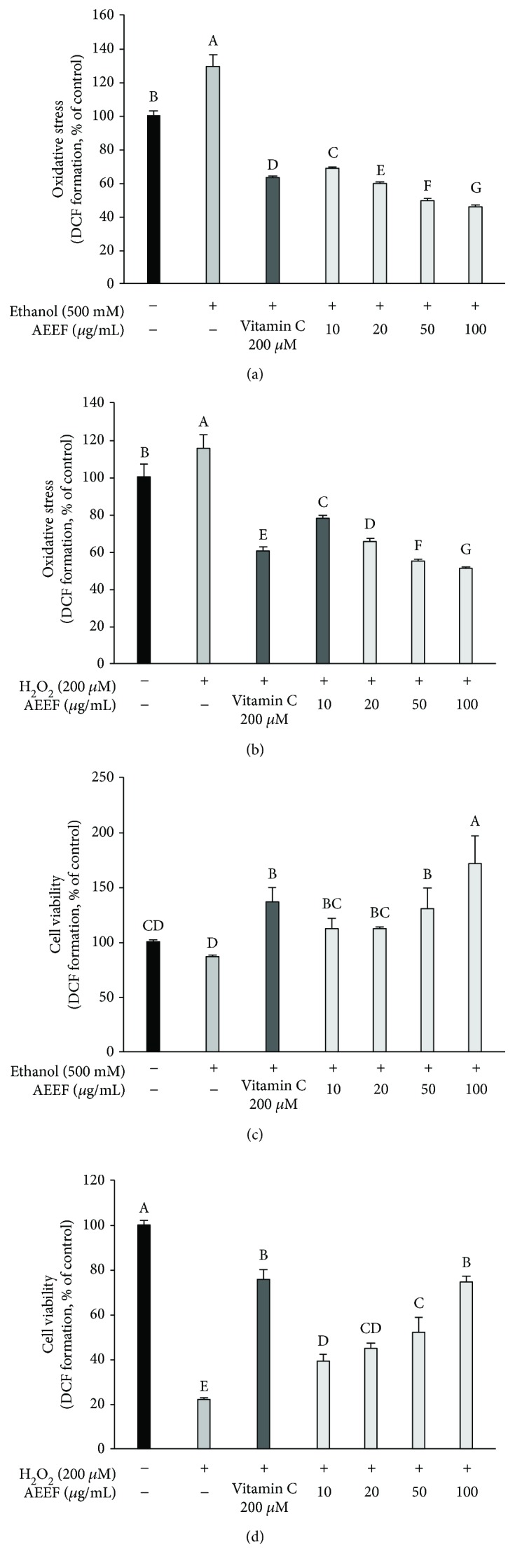
Neuronal cell protective effect of the ethyl acetate fraction from *Aralia elata* (AEEF) in MC-IXC cells. Intracellular ROS content induced by ethanol (a) and H_2_O_2_ (b) and the cell viability against toxicity induced by ethanol (c) and H_2_O_2_ (d). Result expressions are mean and standard deviation (*n* = 5). Statistical differences of data were verified at *p* < 0.05 and, in consequence, indicated as different capital letters in the graph.

DCF-DA and MTT assays were conducted to measure cellular oxidative stress and cell viability against ethanol and H_2_O_2_, respectively. The DCF-DA results (Figures 2(a) and 2(b)) indicated that the ROS content was increased by ethanol (129.28%) and H_2_O_2_ (115.25%) compared to the control group (100.00%), whereas AEEF treatment showed a remarkable reduction in oxidative stress induced by both ethanol and H_2_O_2_ at all concentrations. As the AEEF concentration was increased, the intracellular ROS content induced by both ethanol and H_2_O_2_ was decreased. Treatment with 50 *μ*g/mL (49.46 and 55.15%, respectively) and 100 *μ*g/mL (45.76 and 51.08%, respectively) of AEEF led to a decreased cellular ROS compared to vitamin C (62.97 and 60.36%, respectively).

The results of cell viability to ethanol and H_2_O_2_ are shown in Figures 2(c) and 2(d). The cell viability of the ethanol group (86.49%) and H_2_O_2_ group (22.67%) decreased significantly relative to the control group (100.00%). However, all AEEF groups showed improved cell viability compared to the negative groups. In particular, the 100 *μ*g/mL concentration of AEEF showed similar or higher cell viability (170.95% and 74.48%, ethanol and H_2_O_2_, respectively) compared to the vitamin C group (136.49% and 75.86%, ethanol and H_2_O_2_, respectively).

### 3.2. Effect of AEEF on Behavioral Tests

**Figure 3:**
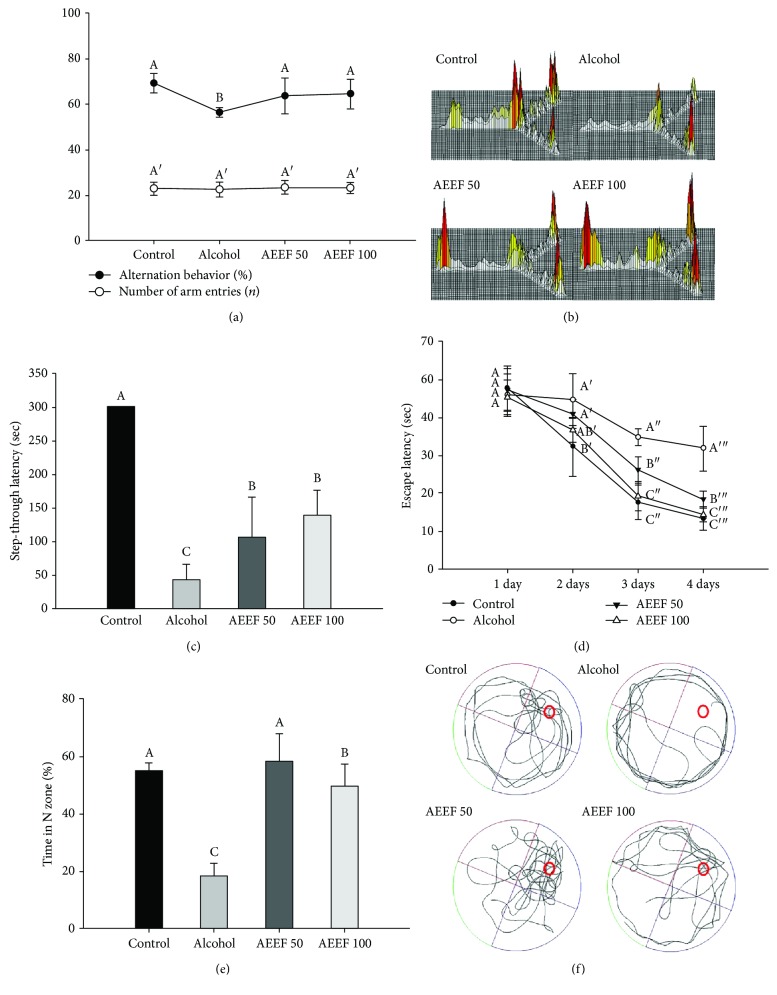
Effect of the ethyl acetate fraction from *Aralia elata* (AEEF) on cognitive function in alcohol-treated mice. Alternation behavior and number of arm entries (a) and path tracing of each group (b) in the Y-maze test; the step-through latency (c) in the passive avoidance test; and escape latency in the training trials (d), time in the N zone of the probe trial (e), and path tracing of the probe trial (f) in the Morris water maze test. Result expressions are mean and standard deviation (*n* = 8). Statistical differences of data were verified at *p* < 0.05 and, in consequence, indicated as different capital letters in the graph.

In Figure 3(a), the results of the Y-maze test showed that the alcohol group (56.19%) had decreased space perceptual ability compared to the control group (68.94%). However, the AEEF 50 (63.43%) and AEEF 100 (64.22%) groups showed statistically similar alternation behaviors as the control group. The number of arm entries was not statistically different among all groups (approximately 22.77 times). In the path tracing results (Figure 3(b)), the alcohol group mainly entered one arm, while the control and AEEF groups showed relatively equal entry into the three arms. To confirm the ameliorating effect on short-term memory impairment, a passive avoidance test was conducted (Figure 3(c)). The step-through latency time of the alcohol group (42.60 sec) was markedly decreased relative to the control group (300.00 sec), whereas the AEEF 50 and 100 groups recovered to 105.40 and 138.40 sec, respectively. A Morris water maze test was conducted to validate learning ability and long-term memory. During the training period, the escape latency time of all groups gradually decreased as the training progressed (Figure 3(d)). Nonetheless, the alcohol group (respective decrease, 30.82%) was significantly different from the other groups (respective decrease: control: 72.15%, AEEF 50: 60.10%, and AEEF 100: 68.65%) in the last training. A probe test was conducted by recording the swimming route of mice without a platform, and the results of recorded path tracing established the cognitive function of the mice through movement. The alcohol group exhibited movement in the whole area (Figure 3(e)) and less retention time (18.11%) in the platform zone (the N zone) compared to the control group (54.76%) (Figure 3(f)). In contrast, there was a considerable improvement of cognitive function from alcohol-induced impairment in the AEEF 50 (58.13%) and AEEF 100 (49.65%) groups.

### 3.3. Ameliorating Effect of AEEF in MDA and SOD Content

**Figure 4:**
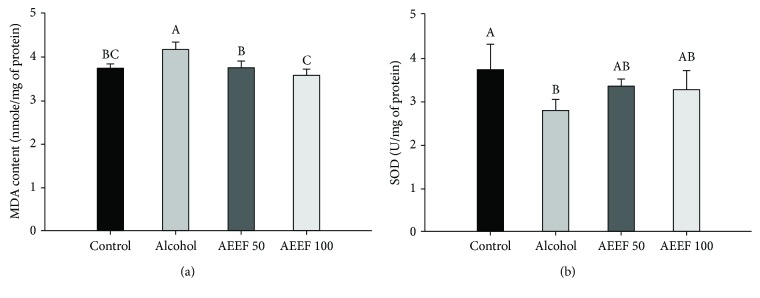
Ameliorating effect of the ethyl acetate fraction from *Aralia elata* (AEEF) on biomarkers. Malondialdehyde (MDA) (a) and superoxide dismutase (SOD) (b) content in the brain homogenate of chronic alcohol-treated mice. Result expressions are mean and standard deviation (*n* = 8). Statistical differences of data were verified at *p* < 0.05 and, in consequence, indicated as different capital letters in the graph.

MDA content was used as a definitive marker of lipid peroxidation. The MDA content of the alcohol group (4.16 nmole/mg of protein) significantly increased compared to that of the control group (3.72 nmole/mg of protein), whereas the AEEF groups (AEEF 50 group, 3.75 nmole/mg of protein) remarkably reduced MDA production in the brain tissue (Figure 4(a)). In particular, the AEEF 100 (3.56 nmole/mg of protein) group effectively inhibited lipid peroxidation compared to the control group. Also, the SOD content of the alcohol group showed decrease (2.77 U/mg of protein) compared with that of the control group (3.71 U/mg of protein) (Figure 4(b)). However, AEEF treatment showed a statistically small increase (AEEF 50 group: 3.33, AEEF 100 group: 3.25 U/mg of protein).

### 3.4. Ameliorating Effect of AEEF in Cholinergic System

**Figure 5:**
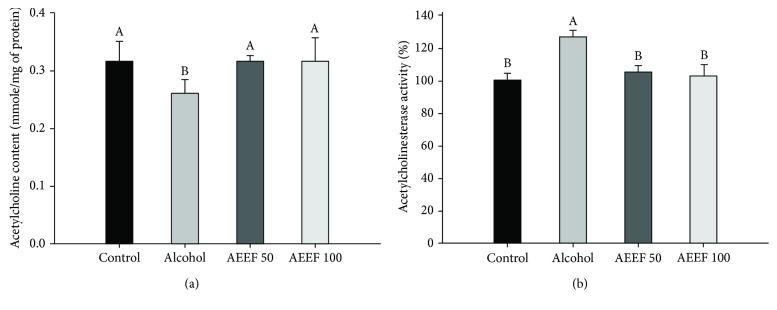
Ameliorating effect of the ethyl acetate fraction from *Aralia elata* (AEEF) on the cholinergic system. Acetylcholinesterase (AChE) activities (a) and acetylcholine (ACh) content (b) in the brain homogenate of chronic alcohol-treated mice. Result expressions are mean and standard deviation (*n* = 8). Statistical differences of data were verified at *p* < 0.05 and, in consequence, indicated as different capital letters in the graph.

To confirm the ameliorating effect of AEEF in the cholinergic system, the content of ACh and activity of AChE were measured (Figure 5). The alcohol group showed excessive AChE activity (126.99%) and lower ACh content (0.26 mmole/mg of protein) in the brain tissue compared to the control group (100.00% and 0.32 mmole/mg of protein, respectively). However, both AEEF treatment groups showed an ameliorated cholinergic system, resulting in similar levels of both AChE activity (104.93 and 102.70%) and ACh content (both 0.30 mmole/mg of protein) compared to the control group.

### 3.5. Effect of AEEF on Mitochondrial Activity

**Figure 6:**
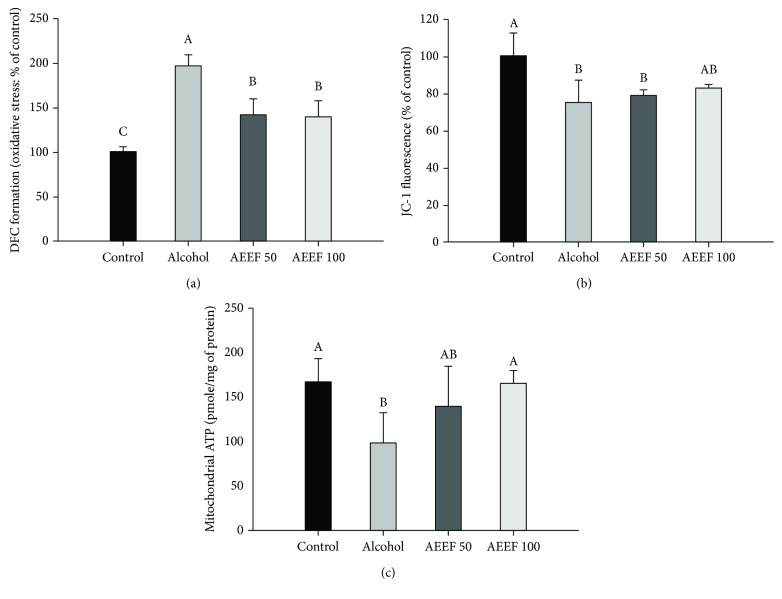
Effect of the ethyl acetate fraction from *Aralia elata* (AEEF) on mitochondrial activity. Mitochondrial reactive oxygen species (ROS) levels (a), mitochondrial membrane potential (MMP) (b), and adenosine triphosphate (ATP) content (c) in isolated mitochondria from the brain homogenate of chronic alcohol-treated mice. Result expressions are mean and standard deviation (*n* = 5). Statistical differences of data were verified at *p* < 0.05 and, in consequence, indicated as different capital letters in the graph.

Mitochondrial activity was evaluated through the ROS level, MMP level, and ATP content to confirm the ameliorating effect of AEEF on alcohol-induced mitochondrial dysfunction. In Figure 6(a), the ROS levels in mitochondria isolated from the brain tissue of the alcohol group (196.65%) had significantly increased, approximately two times more than the control group (100.00%). However, both AEEF groups (141.28 and 139.25%, AEEF 50 and AEEF 100, respectively) showed a substantial decrease in ROS production compared to the alcohol group. The MMP level and ATP content were reduced in the alcohol group (74.75% and 98.89 pmole/mg of protein, respectively) relative to the control group (100.00% and 165.64 pmole/mg of protein, respectively) (Figures 6(b) and 6(c)). Although administration of AEEF 50 (79.06 %) did not significantly improve the MMP levels in alcohol-induced mitochondrial dysfunction, both AEEF groups exhibited elevated ATP content (139.17 and 165.47 pmole/mg of protein, AEEF 50 and AEEF 100, respectively) that was statistically similar to that of the control.

### 3.6. Effect of AEEF on the Apoptotic Signaling Pathway

**Figure 7:**
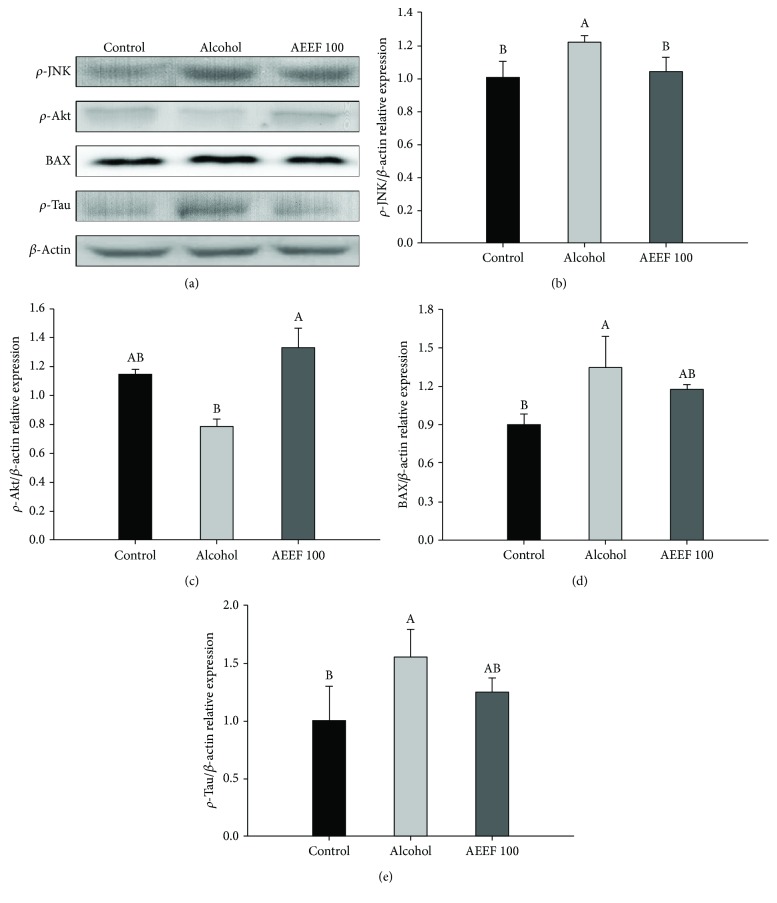
Regulating effect of the ethyl acetate fraction from *Aralia elata* (AEEF) on apoptotic signaling in the brain homogenate of chronic alcohol-treated mice. Relative expression of *p*-JNK (b), *p*-Akt (c), BAX (d), and *p*-Tau (e). Result expressions are mean and standard deviation (*n* = 3). Statistical differences of data were verified at *p* < 0.05 and, in consequence, indicated as different capital letters in the graph.

To confirm the protective effect of AEEF against alcoholic apoptosis, the expression of proteins associated with neuronal cell apoptosis, such as phosphorylated *p*-JNK, *p*-Akt, BAX, and *p*-Tau, was evaluated by western blot analysis. The expression of *p*-JNK as proapoptotic proteins increased 21.52% compared to that of the control group (Figure 7(b)). The AEEF 100 group showed the suppression of *p*-JNK (85.39% compared to the alcohol group). In contrast, the *p*-Akt expression level of the alcohol group was reduced (a decrease of 21.21%) compared with that of the control group (Figure 7(c)). The AEEF 100 group showed the enhancement of *p*-Akt (169.15% compared to the alcohol group). In common with the results of *p*-JNK, the expressions of BAX (an increase of 34.66% compared to the control group) and *p*-Tau (an increase of 55.92% compared to the control group) were significantly upregulated compared to that of the control group (Figures 7(d) and 7(e)). However, the AEEF 100 group showed the suppression of BAX (89.21% compared to the alcohol group) and *p*-Tau (80.50% compared to the alcohol group).

### 3.7. Analysis of Physiological Compounds with UPLC-Q-TOF-MS

**Figure 8:**
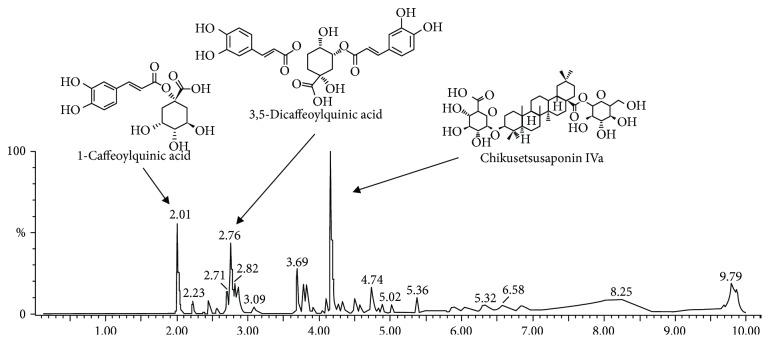
MS^E^ chromatography in the negative ion mode of the ethyl acetate fraction from *Aralia elata* (AEEF) using the UPLC-Q-TOF-MS system.

The major compounds in AEEF were identified using a UPLC-Q-TOF-MS system (Figure 8 and Table 1). The base peaks of main compounds were obtained at 353.09 *m*/*z* (compound A, RT: 2.01 min), 515.12 *m*/*z* (compound B, RT: 2.76 min), and 793.44 *m*/*z* (compound C, RT: 4.16 min) in the negative ion mode. The MS fragmentation ions in the 40-60 eV region showed compound A (191.06 *m*/*z*), compound B (191.06 and 353.09 *m*/*z*), and compound C (631.38 and 569.38 *m*/*z*). The main compounds A, B, and C were identified as 1-caffeoylquinic acid (CQA), 3,5-dicaffeoylquinic acid (3,5-diCQA), and chikusetsusaponin IVa, respectively [24–26].

## 4. Discussion

Alcohol directly causes neurodegeneration and indirectly damages neuronal cells through several pathways such as the production of ROS [4]. ROS is produced in various alcoholic metabolic processes such as catalase and the CYP2E1 system [27]. As with the results of the DCF-DA assay, ROS was increased by treatment with ethanol (Figure 2(a)). Excessive ROS production compared to the antioxidant system which cannot be eradicated in the body leads to lipid peroxidation and mitochondrial dysfunction [7]. Eventually, mitochondria lose the cell survival function, which leads to the apoptosis of neuronal cells. *A. elata* contains various bioactive components, including triterpenoid saponin and the glycosidic form of flavonoids [28]. *Dipsacus asper*, which contains a large amount of triterpene saponin, decreased cytotoxicity in PC12 cells [29]. Also, Ishige et al. reported several protective mechanisms of flavonoid protection against oxidative stress in hippocampal cells [30]. Flavonoids may increase the level of glutathione by removing ROS and inhibiting Ca^2+^ influx. Hence, the above results, namely, the reduction of ROS and increase in cell viability, are also considered to be due to the biological components of *A. elata*.

Previous studies commonly used rodent models fed alcohol for various periods to confirm the harmful effects of chronic alcohol consumption [31, 32]. According to Raghavendra and Kulkarni, Balb/C mice fed ethanol (15%, 2 g/kg) for 24 days showed cognitive deficits and memory impairment [33]. In the mice model of the current study, the behavioral experiment results also showed impairment of cognitive function. Alcohol consumption causes declined judgment and impaired memory. One of the reasons for these defects is the reduction of the brain-derived neurotrophic factor (BDNF), which is a growth factor for neuronal cells. Repeated exposure to alcohol induces a decline in BDNF and inhibition of neurogenesis, which causes difficulties in memory formation [34]. In addition, alcohol damages the hippocampus, which plays an important role in memory formation [35]. This damage induces the loss of hippocampal CA1 and CA3 pyramidal neurons and transient changes in the granule cell number in the DG [36]. Therefore, impairment of the hippocampus leads to trouble in learning ability and space perceptual ability. Also, alcohol causes difficulty in neurotransmission with the diminishment of the acetylcholine level, a neurotransmitter in the cholinergic system [37]. Therefore, in the above behavioral findings, the reduction of cognitive function such as learning ability, space perceptual ability, and memory ability is considered due to the alcohol intake, whereas phenolic compound and saponin activate the survival pathways and expression of BDNF, and they have protective effects in the cognitive function with the protection of the neuronal membrane [38]. Moreover, Socci et al. reported that the administration of antioxidants improves impaired cognitive function [39]. Considering that *A. elata* contains various saponins and phenolic compounds, the administration of AEEF protects the neuronal cell and ameliorates the alcohol-induced impairment of cognitive function.

The process of energy generation occurs naturally in the production of oxidative stress, and it is eliminated by the antioxidant system such as glutathione, catalase, and SOD. SOD reduces oxidative stress by converting superoxide to oxygen or hydrogen peroxide by using the metal ions they have. This property effectively blocks the cascade of cellular oxidation [40]. However, chronic alcohol intake causes damage to antioxidant systems that eradicate these oxidative stresses in the brain tissue [6]. Also, the brain contains rich polyunsaturated fatty acids which are vulnerable to lipid peroxidation by ROS. However, alcohol-induced lipid peroxidation induces the death of neuronal cells and ultimately induces cognitive decline. Therefore, inhibition of lipid peroxidation contributes to cognitive function, and the status of antioxidants is also involved in it [41], whereas AEEF upregulates SOD activity and the inhibition of MDA production in the cerebral tissue. According to Chung and Jung, the ethanol extract of *A. elata* has considerable antioxidant properties and enhances the SOD activity in Sprague Dawley rats treated with benzo(*α*)pyrene [14]. On the other hand, alcohol increases the overgrowth of intestinal gram negative bacteria that produce LPS. This increased LPS produced by intestinal microorganisms stimulates Kupffer cells to produce tumor necrosis factor-alpha (TNF-*α*) [8]. TNF-*α* can enter the blood-brain barrier (BBB) and increase the immune response in the brain. Also, TNF-*α* upregulates the expression of inducible nitric oxide synthase, which creates nitric oxide and various cytokines [23]. However, a previous study showed that the saponins of *A. elata* reduce oxidative stress *via* downregulation of inflammatory signaling such as interleukin- (IL-) 6, IL-1*β*, and TNF-*α* eNOS and iNOS [42]. Consequentially, although alcohol-induced oxidative stress caused neuronal inflammation and damage, cognitive dysfunction could be protected by administrating AEEF containing the various physiologic compounds.

ACh plays an important role in the cholinergic system in cognitive function as a neurotransmitter [43]. ACh is synthesized from acetyl CoA and choline by acetylcholine transferase. The ACh released from synapses stimulates receptors for neurotransmission and opens the ion channels of postsynaptic nerve cells. After the function of the neurotransmitter is completed, ACh is decomposed to acetate and choline by AChE [44]. However, overactivated AChE makes neurotransmission difficult due to the excessive decomposition of ACh [45]. Chronic alcohol consumption causes changes in the cholinergic system [46]. Arendt et al. reported that a decrease in the activity of the cholinergic system was seen over time in Sprague Dawley rats fed 20% (*v*/*v*) ethanol for different periods of time [19]. In particular, significant differences were seen when those rats were treated for 8 weeks. Chronic alcohol consumption showed decreased ACh content and increased AChE activity in almost the whole brain [46]. Also, alcohol promotes lipid peroxidation in the brain tissue. AChE, which has a cell membrane-bound structure, is reported to be more active due to lipid peroxidation [47]. However, it is known that such disorders of the cholinergic system are ameliorated by polyphenolic compounds. The deterpenoids contained in *Aralia cordata*, which belongs to the same family (*Araliaceae*) as *A. elata*, have an inhibitory effect on AChE and a cognitive function improvement effect [48]. In addition, dicaffeoylquinic acids (diCQAs) such as 1,5-diCQA, 3,5-diCQA, and 4,5-diCQA have been reported to help improve the cholinergic system and inhibit the activity of AChE [49]. Although the precise mechanism between the AChE structure and CQA derivatives is unknown, the above results suggest that AEEF helps improve the cholinergic system and improve cognitive function.

Mitochondria which perform a critical role in energy production are an important factor in the apoptosis pathway. Excessive alcohol-induced ROS impairs mitochondrial membrane function through mitochondrial lipid peroxidation and the alteration of mitochondrial membrane properties [7]. These changes include the reduction of MMP in the electron transport chain and decline of energy generation [50]. Moreover, chronic alcohol intake increases not only mitochondrial ROS but also nitric oxide, which is produced by alcohol-induced nitric oxide synthase. This oxidative stress inhibits mitochondrial function by combining with mitochondrial respiratory chain enzymes, and modulates mitochondrial ion channels [51]. Continuous mitochondrial dysfunction becomes the cause of apoptosis progress, and cytochrome c located in mitochondria is released to the cytosol. On the other hands, various phenolic compounds, which act as antioxidants, can reduce mitochondrial oxidative stress [52]. The phenolic compounds protect cells from alcohol-induced cytotoxicity by inhibiting inappropriate changes that can trigger mitochondrial permeability transition and blocking apoptotic pathways [50]. Also, in the results of Zhang et al., the polysaccharide of *A. elata* protects the mitochondria from H_2_O_2_ and inhibits the release of cytochrome c in H9c2 cells [53]. Also, manganese SOD, one of the SOD species, is mainly located in the mitochondrial matrix [40]. Manganese SOD also removes the mitochondrial superoxide, and the mitochondrial ROS result is considered to be associated with previous results and improvement of the SOD level by AEEF (Figure 4(b)). Therefore, these results suggest that AEEF attenuates abnormal energy metabolism through the protection of the mitochondrial function in chronic alcohol-induced neurodegeneration.

Apoptosis is programmed cell death, which is associated with changes to several proteins. The prominent apoptotic route is known as the phosphorylated c-Jun N-terminal kinase (JNK) pathway. JNK is phosphorylated by various stresses, such as UV radiation, inflammatory stimuli, and ROS [54]. Several studies suggest that phosphorylated JNK (*p*-JNK) phosphorylates proapoptotic proteins such as the Bim protein, which activates BAX through the inactivation of Bcl-2 in the mitochondrial apoptosis pathway [55]. Following the cell death signal, BAX translocates to the mitochondria and permeabilizes the mitochondrial membrane by inducing the opening of the mitochondrial voltage-dependent anion channel which reduces the MMP and releases cytochrome c to the cytosol [50]. Released cytochrome c initiates activation of the caspases which induce the apoptosis in the cells. Moreover, phosphorylation of JNK stimulates the phosphorylation of the Tau protein which leads to the death of neuronal cells *via* disintegration of the microtubules and formation of Neurofibrillary tangles [55]. Contrary to this mechanism, activated Akt eliminates the proapoptotic feature of the Bcl-2/BAX complex by inactivating BAD, which is the Bcl-2 associated with the death promoter [56]. As a result of apoptotic signaling, AEEF reduced the expression of *p*-JNK, BAX, and *p*-Tau which cause apoptosis and increased the expression of p-Akt, as an antiapoptotic factor. Similar to these results, Luo et al. reported that saponins of *A. elata* suppress the increased phosphorylation of JNK and inflammatory cytokines in ApoE knockout mice [57]. Also, 3,5-diCQA lowered the ratio of BAX/Bcl in neurons, reducing neuronal cytotoxicity and reducing the release of Ca^2+^ from the cells [58]. Taken together, administration of AEEF prevents apoptosis of neuronal cells with the upregulation of Akt and inhibition of *p*-JNK, which phosphorylates the Tau protein in chronic alcohol-induced neurodegeneration (Figure 7).

CQA and 3,5-diCQA, the main physiologic compounds of AEEH, are known as phenolic compounds commonly found in plants and perform antioxidant and anti-inflammatory activities [59]. In particular, 3,5-diCQA, derived from a (-)-quinic acid and a transcaffeic acid, stimulates the overexpression of phosphoglycerate kinase 1 (PGK1) involved in glycolysis as a glycolytic enzyme that catalyzes the conversion of 1,3-diphosphoglycerate to 3-phosphoglycerate and increases ATP levels in SH-SY5Y neuronal cells [60]. Moreover, 3,5-diCQA has a neuroprotective effect in SH-SY5Y cells and also improves spatial learning and memory in SAMP8 (senescence-accelerated) mice. Also, chikusetsusaponin IVa, an oleanolic acid saponin, is mainly isolated from ginseng and *A. elata* [57]. Moreover, these saponins have been reported to have neuroprotective effects, such as the inhibition of cognitive dysfunction and improvement of the cholinergic system [61]. Chikusetsusaponin IVa also protects neuronal cells against H_2_O_2_-induced oxidative stress *via* activation of SOD and glutathione and upregulates the Sirt1/Foxo3a/Mn-SOD pathway [62]. Therefore, various physiological activities such as the neuroprotective effect and improvement of cognitive function of AEEF have been attributed to its various physiologically active compounds. However, in order to confirm which of these compounds has a high contribution to the physiological activity of AEEF, individual experiments of each compound should be conducted in the future.

## 5. Conclusion

This study suggests that AEEF ameliorates chronic alcohol-induced neurodegeneration with the improvement of cognitive function and protection of brain tissue. Chronic alcohol intake causes oxidative stress and neurodegeneration. However, AEEF exerts a protective effect on neuronal cell survival and significantly improves cognitive functions, such as spatial cognition, memory, and learning ability in ethanol-administrated mice. These ameliorating effects are considered to be due to improvement of the cholinergic and antioxidant system and the reduction of oxidative stress in the brain tissue. In addition, AEEF enhances mitochondrial activity through the inhibition of *p*-JNK expression and oxidative stress and downregulates the apoptotic pathway in neuronal cells. CQA, 3,5-diCQA, and chikusetsusaponin Iva, the main compounds identified in AEEF, may be considered the bioactive components of *A. elata*, and the physiological activity of AEEF is presumed to be due to these compounds. Therefore, *A. elata* has significant potential as a natural agent to ameliorate alcohol-induced neurodegeneration.

## Figures and Tables

**Table 1:** Compounds identified from the ethyl acetate fraction from *Aralia elata* (AEEF).

No.	RT (min)	Parent ion (*m*/*z*)^a^	MS^E^ ions (*m*/*z*)^b^	Compound
1	2.01	353	**191**	1-Caffeoylquinic acid (CQA)
2	2.76	515	353, **191**	3,5-Dicaffeoylquinic acid (3,5-diCQA)
3	4.16	793	**631**, 569, 455	Chikusetsusaponin IVa

^a^Ions are presented in *m*/*z* [M-H]^−^. ^b^Bold indicates the main fragment of MS^E^ ions of each compound.

## Data Availability

The data used to support the findings of this study are available from the corresponding author upon request.

## References

[1] Lieber C. S. (2000). Alcohol and the liver: metabolism of alcohol and its role in hepatic and extrahepatic diseases. *Mount Sinai Journal of Medicine*.

[2] Crews F. T., Buckley T., Dodd P. R. (2005). Alcoholic neurobiology: changes in dependence and recovery. *Alcoholism: Clinical & Experimental Research*.

[3] Butters N. (1985). Alcoholic Korsakoff’s syndrome: some unresolved issues concerning etiology, neuropathology, and cognitive deficits. *Journal of Clinical and Experimental Neuropsychology*.

[4] Haorah J., Ramirez S. H., Floreani N., Gorantla S., Morsey B., Persidsky Y. (2008). Mechanism of alcohol-induced oxidative stress and neuronal injury. *Free Radical Biology & Medicine*.

[5] Sprince H., Parker C. M., Smith G. G., Gonzales L. J. (1975). Protective action of ascorbic acid and sulfur compounds against acetaldehyde toxicity: implications in alcoholism and smoking. *Agents and Actions*.

[6] Lieber C. S. (1997). Ethanol metabolism, cirrhosis and alcoholism. *Clinica Chimica Acta*.

[7] Reddy V. D., Padmavathi P., Kavitha G., Saradamma B., Varadacharyulu N. (2013). Alcohol-induced oxidative/nitrosative stress alters brain mitochondrial membrane properties. *Molecular and Cellular Biochemistry*.

[8] Bode C., Christian Bode J. (2003). Effect of alcohol consumption on the gut. *Best Practice & Research Clinical Gastroenterology*.

[9] Petrasek J., Mandrekar P., Szabo G. (2010). Toll-like receptors in the pathogenesis of alcoholic liver disease. *Gastroenterology Research and Practice*.

[10] Tiwari V., Chopra K. (2013). Protective effect of curcumin against chronic alcohol-induced cognitive deficits and neuroinflammation in the adult rat brain. *Neuroscience*.

[11] Nhiem N. X., Lim H. Y., Kiem P. V. (2011). Oleanane-type triterpene saponins from the bark of *Aralia elata* and their NF-*κ*B inhibition and PPAR activation signal pathway. *Bioorganic & Medicinal Chemistry Letters*.

[12] Yoshikawa M., Murakami T., Harada E., Murakami N., Yamahara J., Matsuda H. (1996). Bioactive saponins and glycosides. VII. On the hypoglycemic principles from the root cortex of *Aralia elata* Seem. : structure related hypoglycemic activity of oleanolic acid oligoglycoside. *Chemical & Pharmaceutical Bulletin*.

[13] Chen Y., Zhao Z., Chen H., Yi T., Qin M., Liang Z. (2015). Chemical differentiation and quality evaluation of commercial Asian and American ginsengs based on a UHPLC-QTOF/MS/MS metabolomics approach. *Phytochemical Analysis*.

[14] Chung C.-K., Jung M.-E. (2003). Ethanol fraction of *Aralia elata Seemann* enhances antioxidant activity and lowers serum lipids in rats when administered with benzo(*α*)pyrene. *Biological & Pharmaceutical Bulletin*.

[15] Hwang K. A., Hwang Y. J., Kim G. R., Choe J. S. (2015). Extracts from *Aralia elata (Miq)* Seem alleviate hepatosteatosis *via* improving hepatic insulin sensitivity. *BMC Complementary and Alternative Medicine*.

[16] Kim J., Park S., Kang J. (2018). Ethyl acetate fraction from persimmon (*Diospyros kaki*) ameliorates cerebral neuronal loss and cognitive deficit *via* the JNK/Akt pathway in TMT-induced mice. *International Journal of Molecular Sciences*.

[17] Bahi A., Tolle V., Fehrentz J. A. (2013). Ghrelin knockout mice show decreased voluntary alcohol consumption and reduced ethanol-induced conditioned place preference. *Peptides*.

[18] Zhao Y. N., Wang F., Fan Y. X., Ping G. F., Yang J. Y., Wu C. F. (2013). Activated microglia are implicated in cognitive deficits, neuronal death, and successful recovery following intermittent ethanol exposure. *Behavioural Brain Research*.

[19] Arendt T., Allen Y., Marchbanks R. M. (1989). Cholinergic system and memory in the rat: effects of chronic ethanol, embryonic basal forebrain brain transplants and excitotoxic lesions of cholinergic basal forebrain projection system. *Neuroscience*.

[20] Kim J. M., Park S. K., Guo T. J. (2016). Anti-amnesic effect of *Dendropanax morbifera via* JNK signaling pathway on cognitive dysfunction in high-fat diet-induced diabetic mice. *Behavioural Brain Research*.

[21] Morris R. (1984). Developments of a water-maze procedure for studying spatial learning in the rat. *Journal of Neuroscience Methods*.

[22] Bradford M. M. (1976). A rapid and sensitive method for the quantitation of microgram quantities of protein utilizing the principle of protein-dye binding. *Analytical Biochemistry*.

[23] Brown M. R., Geddes J. W., Sullivan P. G. (2004). Brain region-specific, age-related, alterations in mitochondrial responses to elevated calcium. *Journal of Bioenergetics and Biomembranes*.

[24] Clifford M. N., Knight S., Kuhnert N. (2005). Discriminating between the six isomers of dicaffeoylquinic acid by LC-MS*^n^*. *Journal of Agricultural and Food Chemistry*.

[25] Weisz G. M., Kammerer D. R., Carle R. (2009). Identification and quantification of phenolic compounds from sunflower (*Helianthus annuus* L.) kernels and shells by HPLC-DAD/ESI-MS^n^. *Food Chemistry*.

[26] Cao Y., Gu C., Zhao F. (2017). Therapeutic effects of *Cyathula officinalis* Kuan and its active fraction on acute blood stasis rat model and identification constituents by HPLC-QTOF/MS/MS. *Pharmacognosy Magazine*.

[27] Koop D. R. (2006). Alcohol metabolism's damaging effects on the cell: a focus on reactive oxygen generation by the enzyme cytochrome P450 2E1. *Alcohol Research & Health*.

[28] Wang Q.-H., Zhang J., Ma X. (2011). A new triterpenoid saponin from the leaves of *Aralia elata*. *Chinese Journal of Natural Medicines*.

[29] Ji D., Wu Y., Zhang B., Zhang C. F., Yang Z. L. (2012). Triterpene saponins from the roots of *Dipsacus asper* and their protective effects against the A*β*_25–35_ induced cytotoxicity in PC12 cells. *Fitoterapia*.

[30] Ishige K., Schubert D., Sagara Y. (2001). Flavonoids protect neuronal cells from oxidative stress by three distinct mechanisms. *Free Radical Biology & Medicine*.

[31] Singh A. K., Jiang Y., Gupta S., Benlhabib E. (2007). Effects of chronic ethanol drinking on the blood–brain barrier and ensuing neuronal toxicity in alcohol-preferring rats subjected to intraperitoneal LPS injection. *Alcohol and Alcoholism*.

[32] Zhou Z., Wang L., Song Z., Saari J. T., McClain C. J., Kang Y. J. (2005). Zinc supplementation prevents alcoholic liver injury in mice through attenuation of oxidative stress. *The American Journal of Pathology*.

[33] Raghavendra V., Kulkarni S. K. (2001). Possible antioxidant mechanism in melatonin reversal of aging and chronic ethanol-induced amnesia in plus-maze and passive avoidance memory tasks. *Free Radical Biology & Medicine*.

[34] Hellmann J., Rommelspacher H., Wernicke C. (2009). Long-term ethanol exposure impairs neuronal differentiation of human neuroblastoma cells involving neurotrophin-mediated intracellular signaling and in particular protein kinase c. *Alcoholism: Clinical and Experimental Research*.

[35] White A. M., Swartzwelder H. S. (2004). Hippocampal function during adolescence: a unique target of ethanol effects. *Annals of the New York Academy of Sciences*.

[36] Puglia M. P., Valenzuela C. F. (2010). Repeated third trimester-equivalent ethanol exposure inhibits long-term potentiation in the hippocampal CA1 region of neonatal rats. *Alcohol*.

[37] Vetreno R. P., Hall J. M., Savage L. M. (2011). Alcohol-related amnesia and dementia: animal models have revealed the contributions of different etiological factors on neuropathology, neurochemical dysfunction and cognitive impairment. *Neurobiology of Learning and Memory*.

[38] Jiang B., Xiong Z., Yang J. (2012). Antidepressant-like effects of ginsenoside Rg1 are due to activation of the BDNF signalling pathway and neurogenesis in the hippocampus. *British Journal of Pharmacology*.

[39] Socci D. J., Crandall B. M., Arendash G. W. (1995). Chronic antioxidant treatment improves the cognitive performance of aged rats. *Brain Research*.

[40] Lebovitz R. M., Zhang H., Vogel H. (1996). Neurodegeneration, myocardial injury, and perinatal death in mitochondrial superoxide dismutase-deficient mice. *Proceedings of the National Academy of Sciences of the United States of America*.

[41] Lovell M. A., Xie C., Markesbery W. R. (2001). Acrolein is increased in Alzheimer’s disease brain and is toxic to primary hippocampal cultures. *Neurobiology of Aging*.

[42] Chen R.-C., Wang J., Yu Y.-L., Sun G.-B., Sun X.-B. (2015). Protective effect of total saponins of *Aralia elata* (Miq) Seem on lipopolysaccharide-induced cardiac dysfunction *via* down-regulation of inflammatory signaling in mice. *RSC Advances*.

[43] Drever B. D., Riedel G., Platt B. (2011). The cholinergic system and hippocampal plasticity. *Behavioural Brain Research*.

[44] Jope R. S. (1979). High affinity choline transport and acetylCoA production in brain and their roles in the regulation of acetylcholine synthesis. *Brain Research Reviews*.

[45] Yoo K. Y., Park S. Y. (2012). Terpenoids as potential anti-Alzheimer’s disease therapeutics. *Molecules*.

[46] Ehrlich D., Pirchl M., Humpel C. (2012). Effects of long-term moderate ethanol and cholesterol on cognition, cholinergic neurons, inflammation, and vascular impairment in rats. *Neuroscience*.

[47] Lasner M., Roth L. G., Chen C. H. (1995). Structure-functional effects of a series of alcohols on acetylcholinesterase-associated membrane-vesicles - elucidation of factors contributing to the alcohol action. *Archives of Biochemistry and Biophysics*.

[48] Jung H. A., Lee E. J., Kim J. S. (2009). Cholinesterase and BACE1 inhibitory diterpenoids from *Aralia cordata*. *Archives of Pharmacal Research*.

[49] Deng J., Qi X. L., Guan Z. Z., Yan X. M., Huang Y., Wang Y. L. (2013). Pretreatment of SH-SY5Y cells with dicaffeoylquinic acids attenuates the reduced expression of nicotinic receptors, elevated level of oxidative stress and enhanced apoptosis caused by *β*-amyloid peptide. *Journal of Pharmacy and Pharmacology*.

[50] Hoek J. B., Cahill A., Pastorino J. G. (2002). Alcohol and mitochondria: a dysfunctional relationship. *Gastroenterology*.

[51] Lacza Z., Kozlov A. V., Pankotai E. (2006). Mitochondria produce reactive nitrogen species *via* an arginine-independent pathway. *Free Radical Research*.

[52] Haraguchi H., Yoshida N., Ishikawa H., Tamura Y., Mizutani K., Kinoshita T. (2000). Protection of mitochondrial functions against oxidative stresses by isoflavans from *Glycyrrhiza glabra*. *Journal of Pharmacy and Pharmacology*.

[53] Zhang J., Wang H., Xue Y., Zheng Q. (2013). Cardioprotective and antioxidant activities of a polysaccharide from the root bark of *Aralia elata* (Miq.) Seem. *Carbohydrate Polymers*.

[54] Dhanasekaran D. N., Reddy E. P. (2008). JNK signaling in apoptosis. *Oncogene*.

[55] Lei K., Davis R. J. (2003). JNK phosphorylation of Bim-related members of the Bcl2 family induces Bax-dependent apoptosis. *Proceedings of the National Academy of Sciences of the United States of America*.

[56] Mehan S., Meena H., Sharma D., Sankhla R. (2011). JNK: a stress-activated protein kinase therapeutic strategies and involvement in Alzheimer’s and various neurodegenerative abnormalities. *Journal of Molecular Neuroscience*.

[57] Luo Y., Dong X., Yu Y., Sun G., Sun X. (2015). Total aralosides of *Aralia elata* (Miq) Seem (TASAES) ameliorate nonalcoholic steatohepatitis by modulating IRE1*α*-mediated JNK and NF-*κ*B pathways in ApoE–/– mice. *Journal of Ethnopharmacology*.

[58] Kim J. Y., Lee H. K., Hwang B. Y., Kim S., Yoo J. K., Seong Y. H. (2012). Neuroprotection of *Ilex latifolia* and caffeoylquinic acid derivatives against excitotoxic and hypoxic damage of cultured rat cortical neurons. *Archives of Pharmacal Research*.

[59] Lee S. G., Lee H., Nam T. G. (2011). Neuroprotective effect of caffeoylquinic acids from *Artemisia princeps* Pampanini against oxidative stress-induced toxicity in PC-12 cells. *Journal of Food Science*.

[60] Han J., Miyamae Y., Shigemori H., Isoda H. (2010). Neuroprotective effect of 3,5-di-O-caffeoylquinic acid on SH-SY5Y cells and senescence-accelerated-prone mice 8 through the up-regulation of phosphoglycerate kinase-1. *Neuroscience*.

[61] Ma H., Han J., Dong Q. (2018). Neuroprotective effect of *Annona glabra* extract against ethanol-induced apoptotic neurodegeneration in neonatal rats. *Journal of Photochemistry and Photobiology B: Biology*.

[62] Wan J., Deng L., Zhang C. (2016). Chikusetsu saponin V attenuates H_2_O_2_-induced oxidative stress in human neuroblastoma SH-SY5Y cells through Sirt1/PGC-1*α*/Mn-SOD signaling pathways. *Canadian Journal of Physiology and Pharmacology*.

